# Pyomyositis Caused by Streptococcus agalactiae: A Case Report and Review of the Literature

**DOI:** 10.7759/cureus.78617

**Published:** 2025-02-06

**Authors:** Masakazu Kakurai, Masahiro Katagiri, Hiroshi Ito, Yoshihiro Moriyama

**Affiliations:** 1 Dermatology, Tsuchiura Kyodo General Hospital, Tsuchiura, JPN; 2 General Internal Medicine, Tokyo Medical University Ibaraki Medical Center, Ami, JPN

**Keywords:** group b streptococcus, infective myositis, pyomyositis, streptococcus agalactiae, tropical pyomyositis

## Abstract

Pyomyositis is a subacute bacterial infection of the skeletal muscle that is more common in the tropics. *Staphylococcus aureus* is the most common pathogen involved in pyomyositis, but *Streptococcus agalactiae* (group B *Streptococcus* (GBS)) can cause pyomyositis. We herein present a case of pyomyositis of the right gluteus maximus caused by GBS bacteremia (primary bacteremia). An 81-year-old man with a history of diabetes mellitus presented with warmth and painful swelling of the right lateral buttock. Computed tomographic (CT) images revealed swelling of the right gluteus maximus without an abscess, but soft tissue inflammation was apparent. The exploratory incision findings excluded necrotizing soft tissue infection. On day 2, two sets of blood cultures taken on admission grew *Streptococcus agalactiae *alone. Thus, pyomyositis caused by GBS was diagnosed. The patient received intravenous ampicillin therapy for two weeks. However, CT images revealed a new area of fluid accumulation in the right gluteus maximus, and no pus was aspirated during ultrasound-guided puncture. Following an additional week of intravenous ampicillin and four weeks of oral amoxicillin, the lesions resolved without surgical debridement. The present case is unique in that the patient developed pyomyositis due to GBS. We summarize and discuss seven cases of GBS pyomyositis, including our case, that have been reported to date.

## Introduction

Pyomyositis is a subacute bacterial infection of the skeletal muscle that is more common in the tropics, but cases have been reported in temperate countries, including the United States [1,2]. The pathogen initially infects the skeletal muscle hematogenously or directly through trauma [1,2]. An overt abscess may form in the skeletal muscle, which can be followed by sepsis, and mortality rates in pyomyositis range from 0.8% to 6% [1,2]. Most cases of pyomyositis are caused by *Staphylococcus aureus* [1,2]. Therefore, limited reports exist of pyomyositis caused by bacteria other than *Staphylococcus aureus*. We herein present a case of pyomyositis of the right gluteus maximus caused by *Streptococcus agalactiae* (group B *Streptococcus* (GBS)) primary bacteremia. We also summarize and discuss seven cases of GBS pyomyositis, including our case, that have been reported to date.

## Case presentation

**Figure 1:**
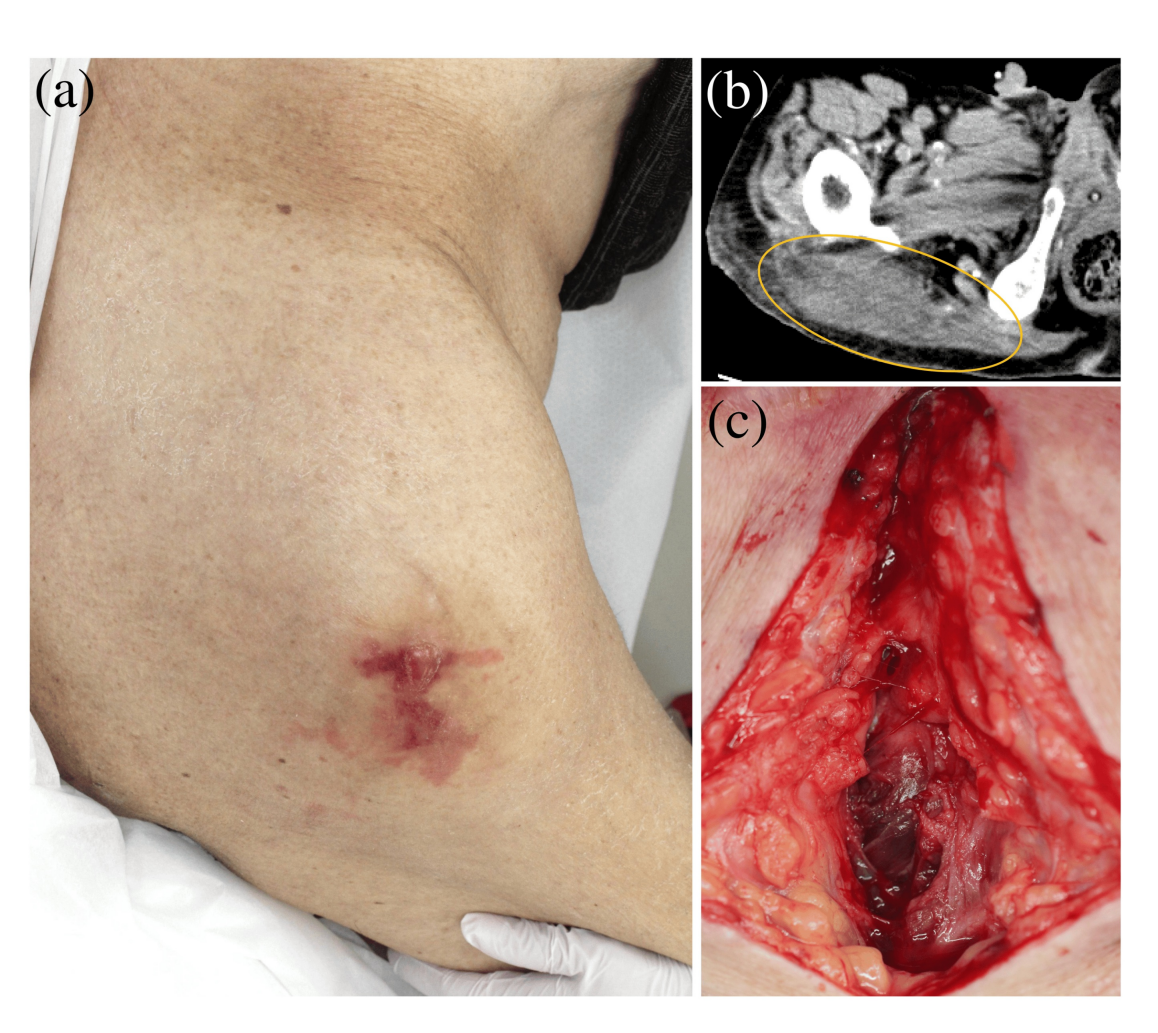
Cutaneous manifestations and CT findings (a) A warm and painful swelling with irregularly shaped erythema and a blister on the right lateral buttock. (b) Swelling of the right gluteus maximus without abscess on CT images (yellow circle). (c) Reddish-brown gluteus maximus without soft tissue necrosis or abscesses. CT: computed tomographic

An 81-year-old Japanese man with a history of diabetes mellitus (DM) presented to the emergency department with right lateral buttock pain and low-grade fever lasting three days. He lived in a geriatric health services facility and performed his daily activities in a wheelchair. On admission, the patient had an axillary body temperature of 37.7℃, a heart rate of 95 beats per minute, a blood pressure of 123/77 mmHg, and a respiratory rate of 22 breaths per minute, without impaired consciousness. Physical examination revealed warmth and painful swelling with irregularly shaped erythema on the right lateral buttock (Figure 1a). The painful swelling was limited to this region, and no pressure ulcer was noted. Blood tests revealed a white blood cell count of 8.10×10^3^/μL, hemoglobin of 10.2 g/dL, platelet count of 13.5×10^4^/μL, blood urea nitrogen of 45 mg/dL, creatinine of 1.80 mg/dL, creatine kinase of 1643 U/L, C-reactive protein (CRP) level of 21.46 mg/dL, and hemoglobin A1c of 5.9% (Table 1). Computed tomographic (CT) images revealed swelling of the right gluteus maximus without an abscess, but soft tissue inflammation was apparent (Figure 1b). No infection in other body regions was suspected based on CT findings. Since these findings suggested pyomyositis or necrotizing soft tissue infection, an exploratory incision was made to the depth of the gluteus maximus, which was reddish-brown, but no soft tissue necrosis or abscesses were observed (Figure 1c). 

**Table 1:** Laboratory data of the blood samples

	Reference value (male)	On arrival	On day 4	On day 9
Aspartate aminotransferase (U/L)	8-38	30	24	14
Alanine aminotransferase (U/L)	4-44	19	14	8
Lactate dehydrogenase (U/L)	124-222	230	243	–
Sodium (mEq/L)	135-147	130	152	139
Potassium (mEq/L)	3.6-5.0	5.0	3.1	4.4
Urea nitrogen (mg/dL)	8-20	45	33	33
Creatinine (mg/dL)	0.61-1.04	1.80	1.63	1.37
Creatine kinase (U/L)	57-197	1643	184	48
C-reactive protein (mg/dL)	0-0.20	21.46	15.72	0.81
White blood cell (/μL)	4,000-9,000	8,100	6,800	5,960
Hemoglobin (g/dL)	14.0-18.0	10.2	11.4	8.5
Platelet (×10^4^/μL)	15.0-35.0	13.5	24.5	23.7
Hemoglobin A1c (%)	4.6-6.2	5.9	–	–

**Figure 2:**
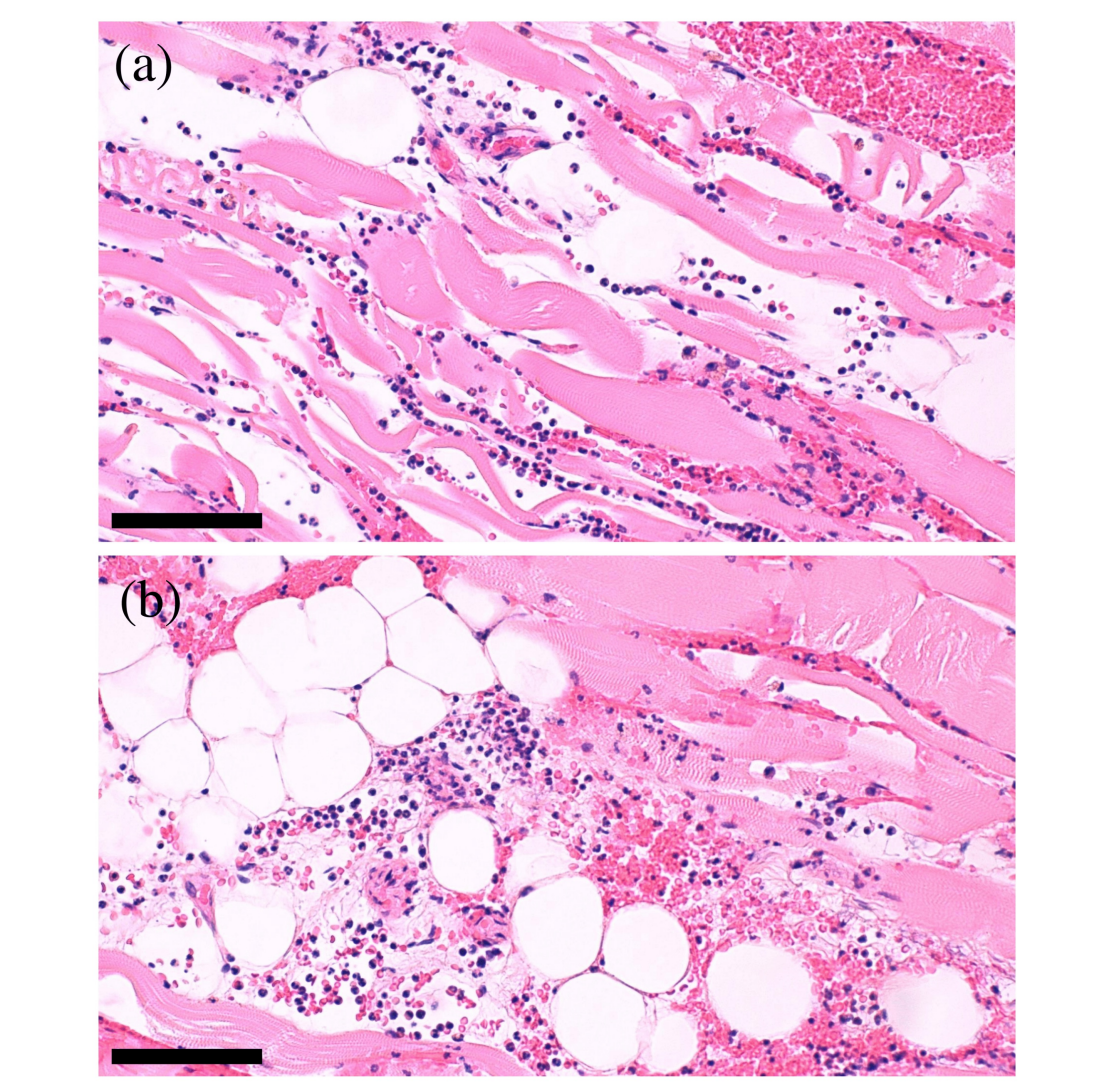
Histological findings (a, b) Multiple infiltrations of neutrophils and red blood cells in the muscle (a) and subcutaneous tissue (b) of the right lateral buttock biopsy (hematoxylin-eosin staining: bar=100 μm, respectively).

Soft tissue biopsy revealed multiple infiltrations of neutrophils in the muscle (Figure 2a) and subcutaneous tissue (Figure 2b) without mononuclear cell infiltrates or thrombosis.

Intravenous ampicillin sodium 6 g daily and clindamycin phosphate 1800 mg daily were initiated. On day 2, two sets of blood cultures taken on admission grew *Streptococcus agalactiae* alone, whereas the urine culture was negative. Taken together, we confirmed the diagnosis of pyomyositis caused by GBS. Clindamycin was discontinued based on the causative bacteria's identification and susceptibility testing results (Table 2).

**Table 2:** Antimicrobial susceptibility of the Streptococcus agalactiae MIC: minimum inhibitory concentration; S: susceptible; R: resistant

Antimicrobial agent	MIC (μg/mL)	MIC interpretation
Penicillin G	0.06	S
Ampicillin	0.12	S
Ampicillin-sulbactam	≤0.25	S
Clindamycin	≤0.12	S
Vancomycin	0.5	S
Levofloxacin	>8	R

**Figure 3:**
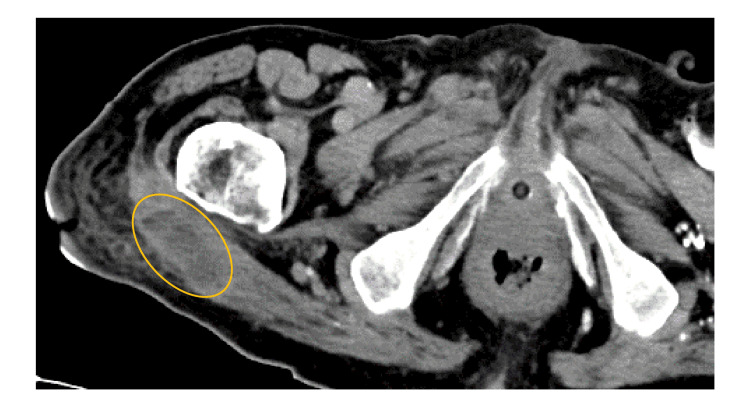
CT findings Defined fluid with a non-enhancing wall (yellow circle) in the swollen right gluteus maximus on contrast-enhanced CT images. CT: computed tomographic

The fever resolved on day 3 and the CRP level decreased; however, the warmth and pain did not improve, and a large amount of exudate continued to drain from the incision site. Contrast-enhanced CT images on day 15 showed a defined area of fluid with a non-enhancing wall accompanied by soft tissue inflammation (Figure 3).

However, no pus was aspirated during ultrasound-guided puncture. Following three weeks of intravenous ampicillin administration, the warmth, pain, and exudate disappeared. Oral amoxicillin was administered for four weeks until CT images confirmed the disappearance of the accumulated fluid. He has been recurrence-free for six months, without antibiotics.

## Discussion

Pyomyositis, formerly known as tropical pyomyositis, is endemic in tropical climates but has recently increased in frequency in temperate countries such as the United States [1,2]. In a previous population-based study in the United States, patients with pyomyositis were predominantly male (63%), and the median age was 44 years, although the pattern was bimodal with a peak incidence in the five- to nine-year age group and another smaller peak in the fifth decade of life [1]. The increased prevalence of risk factors for pyomyositis, including DM, obesity, and human immunodeficiency virus (HIV) infection, may be responsible for the increasing incidence in temperate countries; other risk factors include malnutrition, chronic kidney disease, organ transplantation, and hematological malignancy [1]. The most common pathogen identified in pyomyositis is *Staphylococcus aureus*, accounting for at least 75% of cases in both tropical and temperate countries [1]. Moreover, epidemiological studies of pyomyositis have mainly involved *Staphylococcus aureus*, and whether their findings apply to other bacteria remains unclear. 

Besides *Staphylococcus aureus*, other unusual pathogens implicated in pyomyositis include GBS, *Pseudomonas* species, *Enterococcus*, and *Escherichia coli* [1,2]. To our knowledge, only seven cases of GBS pyomyositis, including ours, have been reported (Table 3) [3-8].

**Table 3:** Reports of pyomyositis caused by Streptococcus agalactiae DM: diabetes mellitus; HIV: human immunodeficiency virus; PBC: primary biliary cholangitis; IV: intravenous; NA: not available

Author/year	Age	Sex	Medical history	Affected muscles	Bacteremia	Antibiotic therapy duration	Course
Back et al., 1990 [3]	54	Male	DM	Rectus abdominis muscles	Positive	5 weeks	Improve
Hull et al., 2008 [8]	11	Male	HIV infection	Left piriformis muscle	Negative	NA	Improve
Panikkath et al., 2016 [5]	58	Male	DM, Hodgkin's lymphoma	Right pectoralis major and minor muscles	Negative	4 weeks	Improve
Unnikrishnan et al., 2018 [4]	73	Female	DM	Right paraspinal muscles	Positive	6 weeks	Improve
Shimizu et al., 2021 [7]	0	Boy	NA	Left obturator internus and quadratus femoris muscles	Positive	5 weeks	Improve
Wang et al., 2023 [6]	74	Female	DM, PBC, breast cancer	Right deltoid, pectoralis, rotator cuff, and lateral chest wall muscles	Negative	8 weeks	Improve
Our case	81	Male	DM, chronic heart failure	Right gluteus maximus muscle	Positive	7 weeks	Improve

GBS is a commensal bacterium of the human genital and gastrointestinal tracts and usually causes infectious diseases in infants and pregnant women [9,10]. However, the incidence of GBS disease among adults (40-79 years) is increasing, often presenting in adults as bacteremia and skin and/or soft tissue infection [10]. In our study, four out of seven cases of pyomyositis occurred in older adults aged 54-81 years (mean: 68 years) [3-6], and the remaining cases were a one-month-old boy whose mother had GBS in her vagina [7] and an 11-year-old boy with HIV infection [8]. All five adult cases had DM [3-6]. As DM is a risk factor for GBS disease among adults [10], it may also be a risk factor for the development of GBS pyomyositis. Affected sites were located on the trunk or extremities other than the head and neck region, and no cases of infection of multiple sites occurred [3-8]. In four cases, including ours, the cause of pyomyositis was presumed to be hematogenous dissemination due to bacteremia [3,4,7]. The remaining cases had negative blood culture results, and the entry of GBS was unknown [5,6,8]. 

Although the duration of treatment for pyomyositis varies depending on the type of bacteria and the condition of the host, one to two weeks of intravenous therapy followed by approximately four weeks of oral treatment is recommended [2]. While many clinicians prefer surgical debridement of abscesses, others prefer a less invasive approach, opting for an image-guided drainage [2]. One of the most common complications of pyomyositis is sepsis, with relatively rare complications such as meningitis, spinal abscess, cardiac tamponade, arthritis, osteomyelitis, and compartment syndrome [2]. Mortality rates in pyomyositis range from 0.8% to 6%, suggesting a good prognosis [2]. In our literature review, all cases of GBS pyomyositis improved with long-term antibiotic therapy (mean: 5.8 weeks; range: 4-8 weeks). Among the cases where an abscess occurred, most involved image-guided drainage [3-5], and one included surgical debridement [6]. None of the cases had the abovementioned complications, and all cases showed improvement. Therefore, GBS pyomyositis may have a good prognosis, but this may be due to the fact that all cases were promptly diagnosed and received appropriate treatment.

Pyomyositis is a subacute inflammatory process that can be divided into three stages [2,6]. The first stage begins with dull muscle pain and low-grade fever for one to three weeks, but accumulation of pus at the site is not apparent. The second stage involves fever, severe muscle pain, and increased swelling. During this stage, an abscess develops in the muscle, which is histologically described as containing numerous neutrophils, perivascular mononuclear cell infiltrates, and thrombosis. Finally, sepsis occurs in the third stage. Our patient had a mild fever with no severe pain or abscess on admission. However, fluid accumulated in the muscle during hospitalization that could not be drained through puncture. Therefore, our patient may have been transitional (from stage 1 to 2), a stage that has not been previously described in the literature. 

The clinical characteristics and treatment of GBS pyomyositis are not fully understood due to the low number of reported cases. Therefore, further accumulation of GBS pyomyositis cases is warranted to help clarify the pathogenesis, clinical characteristics, treatment methods (surgical debridement vs. image-guided drainage), and appropriate duration of antibiotic therapy.

## Conclusions

Pyomyositis may be accompanied by soft tissue inflammation without intramuscular abscesses on CT images at the first visit, as in our case, and must be differentiated from necrotizing soft tissue infection. In addition, patients with pyomyositis generally have a good prognosis, but it can lead to sepsis. Therefore, it is important to exclude necrotizing soft tissue infection by performing an exploratory incision, make an early diagnosis, and provide appropriate treatment of pyomyositis.

Additionally, the present case is unique in that the patient developed pyomyositis due to GBS, which is an unusual pathogen for pyomyositis. To our knowledge, seven cases of GBS pyomyositis, including ours, have been reported. All five adult cases had DM, which may be a risk factor for the development of GBS pyomyositis. All cases of GBS pyomyositis were treated with long-term antibiotic therapy and had a good prognosis without complications. Further accumulation of cases of GBS pyomyositis and large case series are necessary to advance the understanding of the epidemiology and clinical characteristics of GBS pyomyositis and optimize appropriate treatment strategies.
